# Investigating Sensitivity to Shared Information and Personal Experience in Children’s Use of Majority Information

**DOI:** 10.1162/opmi_a_00182

**Published:** 2025-02-08

**Authors:** Rebekah A. Gelpí, Kay Otsubo, Amy Whalen, Daphna Buchsbaum

**Affiliations:** Department of Psychology, University of Toronto, Toronto, Canada; Schwartz Reisman Institute for Technology and Society, University of Toronto, Toronto, Canada; Roslin Institute, Midlothian, UK; Department of Cognitive and Psychological Sciences, Brown University, Providence, RI, USA

**Keywords:** social learning, testimony, selective trust, conformity bias, cognitive development

## Abstract

Children and adults alike rely on others to learn about the world, but also need to be able to determine the strength of both their own evidence as well as the evidence that other people provide, particularly when different sources of information disagree. For example, if two informants agree on a belief but share the same evidence, their testimony is statistically dependent on each other, and may be weaker evidence for that belief than two informants who draw on different pieces of evidence to support that belief. Across three experiments (total *N* = 492), we examine how 4- and 5-year-old children evaluate statistical dependency on a task where they must determine which of two jars that toys were drawn from. A majority of informants, whose testimony could draw from the same evidence or different evidence, always endorsed one jar. Then, children were presented with a dissenting informant or their own personal data that was consistent with the other jar. Children showed no sensitivity to statistical dependency, choosing the majority with equal probability regardless of the independence of their testimony, but also systematically overweighted their own personal data, endorsing the jar consistent with their own evidence more often than would be predicted by an optimal Bayesian model. In contrast, children made choices consistent with this model on a similar task in which the data was presented to children without testimony. Our findings suggest that young children treat majorities as broadly informative, but that the challenges of inferring others’ experiences may lead them to rely on concrete, visible evidence when it is available.

## INTRODUCTION

Imagine that you are at a carnival with some friends, and you are trying to find out which booth has more of a specific giant panda prize you are looking for. If two friends told you that they each saw someone win a panda at the red booth, and one friend told you that they saw someone win a panda at the yellow booth, you might be more likely to go to the red booth. We frequently rely on information that we get from others, particularly in situations where we have little or incomplete information ourselves, and our use of information we get from others is not simply a helpful tool for day-to-day dilemmas, but a characteristic thought to underpin our cultural evolution and success as a species (Boyd et al., 2011). We seek out and integrate social information with our own understanding strategically, attending to characteristics such as our own uncertainty, the identity of an informant, or the frequency with which we observe a piece of information (Kendal et al., 2018; Rendell et al., 2011).

Specifically, we sometimes encounter situations where our social informants disagree, as in the example provided above. In these situations, we may choose to rely on the opinion provided by a majority of people. Intuitively, a strategy of heeding the information from a majority is often a sensible one, as it facilitates the cultural transmission of adaptive behaviors (Boyd & Richerson, 1985; Henrich & Boyd, 1998), and numerous studies have argued that many species, including humans, attend to the actions of a majority when judging how to act themselves (Efferson et al., 2008; Laland, 2004; Pike & Laland, 2010).

However, majorities can also be incorrect or have incomplete information, so we also need to be able to evaluate the quality of the information we receive and understand how others formed their beliefs. One potential way in which we may be led astray by naively using the size of a majority to determine whether to endorse the same belief is by assuming that every individual within the majority independently collected their own private data to support their conclusion, rather than basing their views on a smaller number of shared sources (van Leeuwen et al., 2015; Whalen et al., 2018). However, in many real-world situations members of a group share or observe the same pieces of data with each other. For example, returning to the example of the carnival, if both of your friends who told you that they saw someone at the red booth win the giant panda prize you were looking for were actually observing the same person, then their belief is based on the same shared piece of evidence, and there is now an equal amount of evidence for giant panda prizes at each booth, even if the number of people endorsing the red booth is higher. When members of a majority agree because they are obtaining evidence from the same shared source, each individual member is providing less evidence than if their information was supported by independent, converging data.

In addition, in many real-world cases, while we cannot directly observe all of the data, we can often collect some information ourselves, which must be integrated with the information we receive from others. For example, imagine recalling that you previously obtained the prize you wanted from the red booth, while two other friends say that the yellow booth is more likely to carry the prize. In this situation, the information you have is relevant, but not conclusive—you do not have deterministic evidence for which booth contains more of the prize (and neither do your friends)—so you must determine both the quality of the information provided by your friends, as well as the value of your own conflicting experience in light of their testimony.

Learning how to evaluate the quality of social testimony is particularly important for children, who know less than adults and as such must rely comparatively more on learning about the world from the testimony of others. From an early age, children have a robust tendency to trust what they are told (Jaswal et al., 2010), but very quickly learn to develop sophisticated social learning strategies to judge the quality of their prospective informants and the data that they provide (for reviews, see Harris et al., 2018; Mills, 2013).

In this set of studies, we focus on how children evaluate informant testimony and their own evidence in a scenario where everyone has incomplete information. As mentioned above, a bias towards conforming to a majority’s belief is often a useful heuristic for the quality of the majority informants’ information, since broadly shared beliefs and behaviors are typically well-adapted to one’s environment. However, if members of a majority are basing their beliefs on a small number of primary sources, this heuristic overestimates the informativeness of the majority’s views. We aim to examine whether children’s reasoning about information from majorities incorporates the statistical dependence of these shared views, and how children integrate this reasoning with their reasoning about information they personally observe.

### Adults’ Reasoning About Majorities

In general, adults are capable of sensibly reasoning about the quality of information provided by a majority, at least when the informants’ sources of information are clear and salient (though they may be influenced by majority size under other circumstances, as discussed below). For example, in experiments conducted by Whalen et al. (2018), adults were presented with a situation where the participant, as well as all of the informants had incomplete personal information, and where they had to either endorse a majority’s belief or a dissenting minority’s belief. When individuals in the majority each collected their data independently, adults preferred to endorse the majority, but correctly showed no preference when the majority had all based their opinion on a single piece of shared data. In addition, adults properly weighed their own personal data and knowledge as comparable to, but not significantly more than that of a third party. Taken together, adults in this experiment displayed a sensitivity to statistical dependency between informants when socially learning from a majority, and could appropriately weigh the quality of information from multiple sources, including their own knowledge as well as hearsay.

However, under some circumstances, adults do appear to be susceptible to an “illusion of consensus”, treating information from multiple people who rely on information from a single shared source to be as reliable as information from multiple people who each rely on their own independent source (Alister et al., 2022; Yousif et al., 2019). In addition, people can sometimes experience “information cascades”—situations in which the presence of an early emerging majority opinion leads people to discount their own private data and endorse the majority belief, creating a cascading perception of increasing consensus even when successive new endorsements are in fact based only on the previous endorsements and not on new private data (Anderson & Holt, 1997). These dynamics have been argued to underlie the transmission of fads, cultural changes, distorted information, and misinformation (Bikhchandani et al., 1992, 1998; Horta Ribeiro et al., 2019).

Nevertheless, adults also consider testimony from informants who overhear one another’s testimony before they provide their own testimony less informative than conflicting testimony from informants who state their beliefs privately (Einav, 2018). Likewise, when the statistical dependence between informants is highlighted, people treat information obtained from a “false consensus” as less strong than that of a “true consensus” of independent sources (Desai et al., 2022), again suggesting that adults understand that statistically dependent evidence is less informative, even if the degree of independence of sources is more difficult for them to establish in some circumstances.

### The Development of Children’s Selectivity in Social Learning

An important milestone in young children’s social learning is the development of selectivity in their trust and endorsement of others’ information. Children as young as 3 years old evaluate the reliability of individual informants when considering who to learn from, preferring to learn from those who are knowledgeable, accurate, or reliable (Fusaro & Harris, 2008; Jaswal & Neely, 2006; Koenig et al., 2004; Koenig & Harris, 2005) and informants with domain-specific expertise (Aguiar et al., 2012; Boseovski & Thurman, 2014; Kushnir et al., 2013). However, as Koenig and Harris (2007) note, young children’s assessments of the reliability of testimony may often reflect an assessment of the informant themselves, rather than of the quality of their information. As a result, it is less clear whether children at this age can selectively learn based on the quality of the information itself, and in particular, whether children have a sensitivity to statistical dependencies between informants.

#### Selective Trust in Majorities.

Similarly to adults, children understand that it is often sensible to attend and conform to the endorsements of a majority, particularly in ambiguous or normative settings such as labelling novel objects (Corriveau et al., 2009; Fusaro & Harris, 2008; Pham & Buchsbaum, 2020) or when learning strategies on causal tasks (Pham & Buchsbaum, 2020; Wilks et al., 2015). They sometimes even do so in unambiguous settings where they have complete information, and know the majority is incorrect, such as a task where children judged which image was larger (Corriveau & Harris, 2010; Haun & Tomasello, 2011), suggesting children may at times display a conformity bias.

However, other studies have indicated that children endorse a majority less often when it is shown to have information of lower quality. For example, 4-year-old children have been observed to go against the majority when the majority group is unsuccessful in reaching an apparent goal (Wilks et al., 2015), provides implausible functions for a novel object (Schillaci & Kelemen, 2014), has no privileged knowledge (Einav, 2014), and displays lower certainty (Pham & Buchsbaum, 2020). Children are also less likely to endorse the majority belief when they can directly observe their own conflicting data (Pham & Buchsbaum, 2020) or when there is variation or disagreement within a population (Morgan et al., 2015), and at least some children prefer to learn from an expert over a non-expert majority (Burdett et al., 2016). Thus, young children do not always exhibit a conformity bias, and particularly appear to be less likely to conform to a majority in situations where the majority’s information is of lower quality.

#### Children’s Understanding of Statistical Dependence.

Another important element to children’s developing capacities to evaluate the quality of social information is their reasoning about the sources of individuals’ knowledge. By 3 years of age, children understand that perceptual experience is a source of others’ knowledge (Pillow, 1989), and with age, increasingly expect informants’ knowledge to be justified, i.e., an informant visually checking a box before claiming what is inside (Butler et al., 2018, 2020). However, we are often told by others what they think without seeing the data they used to form their opinion. Accurately evaluating the quality of this social information requires not only that children understand the testimony another person provides, but also that children draw inferences about the unseen data or primary sources used to formulate this knowledge. Although most children can explicitly reason about others’ mental states by 4 to 5 years of age (Wellman et al., 2001; Wellman & Liu, 2004; Wimmer & Perner, 1983), understanding that multiple informants’ testimony is statistically dependent due to sharing a single unseen data point may require a more complex theory of mind.

The fact that 4- and 5-year-old children do not always exhibit conformity with majorities suggests that children are sensitive to a number of cues to information quality. However, the evidence regarding how children evaluate the epistemic value of statistically dependent information suggests that this is a more challenging task. For example, Einav (2018) found that while 8- and 9-year-olds preferred to endorse the choice of a group where members all independently came to the same conclusion rather than a group where members looked at each other’s answers before responding, 5-year-olds exhibited the reverse pattern, endorsing the dependent group more often than the independent group. Here, young children may have reasoned that copying was itself a sign of the reliability or prestige of the first respondent (e.g., Chudek et al., 2012), while not considering the potential epistemic value of independent, converging answers. Similarly, Aboody et al. (2022) examined children’s endorsements of testimony from informants who themselves based their testimony on differing numbers of primary informants. 6-year-olds, but not 5-year-olds, inferred that a group of individuals basing their testimony (which direction a hamster ran away) on three primary sources was more likely to be correct than a group whose testimony was supported by only one primary source.

Nevertheless, there is also evidence that children younger than 5 recognize that dependent informants provide less information when the strength of the evidence available to the groups is clearer. For example, Hu et al. (2015) found that 3- to 5-year-olds choose an option endorsed by a group where every individual has direct perceptual access to the options, rather than the option endorsed by an equally sized group where only one individual has direct access and other members copy the first, suggesting that children in this age range may be in the midst of developing an understanding of statistical dependence.

#### Children’s Integration of Conflicting Experience.

As mentioned previously, social learning also requires the understanding that one’s own experiences may conflict with those of others—and that others’ testimony may sometimes be more informative, particularly when one’s own knowledge about a situation is incomplete. By 4 years of age, children develop a sense of skeptical trust, relying on their own conflicting observations when an informant was previously inaccurate but extending trust to a previously credible informant (Clément et al., 2004; Ma & Ganea, 2010), as well as balancing these considerations with the statistical patterns of data they observe, and an informant’s confidence in their own testimony (Bridgers et al., 2016; Hermansen et al., 2021). Thus, although children may rely heavily on testimony, they evaluate it critically, considering the quality of their own evidence as well.

Nevertheless, particularly when data is ambiguous, children sometimes display a “self-agency bias” (Kushnir et al., 2009; Tong et al., 2023; Yuniarto et al., 2020), privileging their own observations more heavily and learning from them more effectively than those of others. Thus, when faced with a conflict between their own observations and other’s testimony, such a bias could potentially lead children to discount valuable social information.

In other cases, children’s preference for their own data may reflect a recognition that they have received sufficiently strong information about the situation and do not require additional testimony. For example, 4-year-old children were more likely to reject testimony from a knowledgeable informant when they were presented with more conclusive evidence that conflicted with the testimony than when they were presented with ambiguous evidence, i.e., probabilistic evidence that conflicted with the testimony (Bridgers et al., 2016).

In summary, the current literature suggests that 4- and 5-year-old children’s social learning is selective, allowing them to integrate information from multiple sources including themselves, and that children are at least sometimes able to understand that informants provide information of varying qualities, even when this conflicts with other characteristics that children rely upon, such as the presence of a majority endorsing an option.

Here, we focus on how young children evaluate conflicting information across two potential cues to the strength of the evidence they observe: whether the testimony they receive from the majority of informants is statistically dependent or independent, and whether the conflicting information takes the form of a dissenting informant or the child’s own conflicting experience. Given that prior work suggests that children are more likely to integrate others’ testimony when they perceive that their own information is incomplete or ambiguous, but not when evidence is more conclusive (e.g., Bridgers et al., 2016) and that young children effectively reason about the statistics of probabilistic outcomes in sampling tasks (Denison, Bonawitz, et al., 2013; Denison et al., 2006; Kushnir & Gopnik, 2005), we anticipate that if children younger than 6 years old can reason about statistical dependency in the context of integrating their own evidence with conflicting majority testimony, they would be most likely to do so in a context where their evidence is ambiguous or incomplete. Thus, we use a probabilistic sampling task to assess how children integrate information from informants whose evidence carries varying strength.

If 4- and 5-year-olds reason about statistical dependency in the same way that adults do (e.g., Desai et al., 2022; Whalen et al., 2018) we predict that children should perceive a majority whose information is based on the same shared evidence to be less convincing, and endorse this majority’s opinion less than a majority whose evidence was collected independently. On the other hand, if children do not show this sensitivity to statistical dependence, we expect that children should endorse both majorities equally.

Across 3 experiments, we therefore investigate how 4- and 5-year-old children evaluate testimony from a majority that either independently collects private data or shares a single piece of evidence, and how they integrate this information with dissenting information provided either by a single dissenting informant or by conflicting data the child is provided directly. In Experiments 1 and 2, we test whether children are sensitive to statistical dependence, treating information from a majority differently when data is either shared or collected independently, and in Experiment 3, we introduce a control condition testing children’s ability to reason about statistical samples. We also compare children’s responses to the *a priori* predictions of a family of Bayesian models that make concrete predictions about how an ideal observer would evaluate this evidence, and to adults, whose behaviour is effectively captured by this model.

## BAYESIAN MODEL OF LEARNING FROM INDEPENDENT AND DEPENDENT INFORMANTS

To first develop concrete predictions about how a child who displayed sensitivity to statistical dependence should evaluate evidence from the majority, we adapt a Bayesian model of social learning from Whalen et al. (2018) that captures how a learner should integrate testimony provided by majorities with different sources of information—the majority’s evidence is either shared (e.g., they all observed the same winner at the red booth) or independent (e.g., each majority member saw a different person win a panda)—with the learner’s own data. The predictions of this simple, parameter-free model closely resembled adults’ judgments on the same tasks, suggesting that when the statistical dependence and independence of social testimony is clear, adults’ choices reflected normative considerations of the strength of the evidence.

Our experimental design resembles the classic “ball and urn” scenario conducted by Anderson and Holt (1997). This paradigm allows us to test a learner’s use of statistical evidence to evaluate different hypotheses, and closely resembles similar tasks conducted with children (Denison, Bonawitz, et al., 2013; Denison et al., 2006) and infants (Denison, Reed, & Xu, 2013; Denison & Xu, 2010). In our task, an experimenter has two jars of balls, one filled mostly with red balls but with a small number of yellow balls, and one filled mostly with yellow balls and a small number of red balls. The experimenter hides the jars from view, then picks one jar to sample from. The experimenter then shows each of three informants their own ball, out of view of the participant. The informants then state which jar they think was chosen. The experimenter also shows a ball to the participant, and then asks which jar they think all of the balls were drawn from. If all three informants indicate their belief that the samples are from the mostly red jar, but the participant receives a yellow ball, which jar should the participant pick?

This thought experiment can be modelled as a Bayesian reasoning problem. The participant collects personal data *e*—in this case, a yellow ball—that provides evidence as to the identity of the jar. In addition, they also receive testimony from multiple informants *t*_1_, …, *t*_*n*_ who each collect their own data, *d*_1_, …, *d*_*n*_. In this case, 3 informants stated they believed their ball was drawn from the mostly red jar. Assuming that the experimenter randomly and independently sampled each ball from the jar, the participant can then evaluate a potential hypothesis about the source of the sampled balls, *h*, using Bayes’ rule:

$$p\left(h|e,t_{1},\ldots ,t_{n}\right)\propto p\left(t_{1},\ldots ,t_{n}|h\right)p\left(e|h\right)p\left(h\right)$$ (1)

In this equation, the posterior probability of the hypothesis given the data available to the participant, *p*(*h*∣*e*, *t*_1_, …, *t*_*n*_), is proportional to the likelihood of the informants’ testimony given the hypothesis, *p*(*t*_1_, …, *t*_*n*_∣*h*), multiplied by the likelihood of the participant receiving their own personal data given the hypothesis, *p*(*e*∣*h*), and the prior probability of the hypothesis, *p*(*h*). Hereafter, *h*_1_ = mostly red jar, *h*_2_ = mostly yellow jar, *d*_1_ = red ball, *d*_2_ = yellow ball.

If each informant received their own private independent sample from the bag, the probability of a particular series of testimony is equivalent to the product of the probability of each individual piece of testimony:

$$p\left(t_{1},\ldots ,t_{n}|h\right)=\prod_{i=1}^{n}p\left(t_{i}|h\right)$$ (2)

The testimony of each informant is based on their own respective sample, *d*_*i*_, so *p*(*t*_*i*_∣*h*) is obtained by marginalizing over *d*_*i*_:

$$p\left(t_{i}|h\right)=\sum \left(d_{i}\right)p\left(d_{i}|h\right)p\left(t_{i}|d_{i}\right)$$ (3)

where *p*(*t*_*i*_∣*d*_*i*_) is the probability that the informant produces testimony *t*_*i*_ after observing their own private data, *d*_*i*_.

Alternatively, the three informants could have shared a single ball when each making their decision to endorse the red jar. In this case, the informants are basing their testimony on a shared single piece of data, denoted as *d*′. As such, the probability of a series of testimony is obtained by marginalizing over the shared single piece of data:

$$p\left(t_{1},\ldots ,t_{n}|h\right)=\sum_{d^{\prime }}p\left(d^{\prime }|h\right)\prod_{i}p\left(t_{i}|d\right)$$ (4)

This model represents both the participant and the informants as normative learners who make the best possible inference from the data available. One way such a learner could reason about their data would be to deterministically choose the hypothesis (jar) with the highest probability of generating the evidence (their ball). For example, an informant who saw a red ball would always endorse the red jar: *p*(*t*_*i*_ = *h*_1_∣*d*_1_) = 1. This strategy, known as maximization, has been observed among children under the age of 5 (Derks & Paclisanu, 1967; Jones & Liverant, 1960), particularly in language learning paradigms (Hudson Kam & Newport, 2005, 2009). Adults also sometimes display maximization behavior in a number of economic game settings, particularly with higher levels of feedback and training (e.g., Schulze et al., 2020; Shanks et al., 2002).

However, in many situations both children and adults as well as nonhuman animals have been found to instead probability match (e.g., Denison, Bonawitz, et al., 2013; Vulkan, 2000), choosing a hypothesis in proportion to how likely they think it is to be correct, based on their own personal data and prior beliefs about the world: *p*(*t*_*i*_ = *h*_1_∣*d*_1_) ∝ *p*(*d*_1_∣*h*_1_)*p*(*h*_1_). For example, if an informant states that they believe their ball was drawn from the mostly red jar, the learner should assume that this is because the informant was more likely to have seen a red ball, given the prior probabilities of drawing a red ball from the mostly red versus mostly yellow jars. Adults’ choices on a similar task most closely matched the predictions of the probability matching model (Whalen et al., 2018); given the mixed evidence regarding children’s strategy use, we evaluate children’s choices relative to the predictions of both the maximizing and probability matching models.

Regardless of whether informants probability match or maximize, the models make different predictions based on how the informants collected their personal data and the statistical dependency of that information (for full model predictions, see Figures 2, 4, and 6). For example, it supports that it is rational to follow the majority when members have independently collected their own private data, as it increases the probability of the majority’s hypothesis. However, if the majority shares a single piece of data, then their evidence should be considered no more convincing than the participant’s own conflicting data.

In the current study, we run three behavioral experiments to evaluate how 4- and 5-year-old children decide whose beliefs to endorse when receiving both majority testimony and conflicting data either directly, or through a dissenting informant. By examining preschoolers’ judgments, we can assess whether they are similarly sensitive to statistical dependency as adults are, or if they weigh evidence received from testimony differently, for instance by displaying a stronger conformity bias. We can then compare their performance to that of our Bayesian models to predict what pieces of evidence children believe have a relatively greater or lower quality of information.

## EXPERIMENT 1

In Experiment 1, we aimed to test the predictions of our Bayesian models by presenting children with a scenario in which they must integrate testimony from informants whose data varies in the degree to which it is independent, as well as conflicting information they receive either directly, or through a dissenting informant. Children were shown a video about two jars with differing proportions of red and yellow balls and were asked to guess which jar was being sampled from, given the testimony of informants who received a ball from the chosen jar and dissenting information. The first 2 or 3 (depending on condition) informants endorsed the same jar and made up the majority group. The members of the majority either received independent samples from the jar, each receiving their own ball, or shared a single sample (ball) amongst the group, making their testimony statistically dependent. A dissenting sample was either provided to an additional informant, or presented to the child as the child’s own sample. Thus, we conducted a 2 × 2 design, where there were 2 majority ball conditions—independent and shared, and 2 dissenting information conditions—informant and own ball.

Here, we predicted that if children were sensitive to statistical dependency to the same degree as adults, then children would respond as anticipated by the Bayesian rational model (Figure 2). Namely, the model predicts that children will be more likely to endorse the majority when the majority’s data was collected independently, because there are a larger number of independent sources. When the majority’s data is shared, the models predict at or near chance choices, because most likely one red ball and one yellow ball have been sampled. Specifically, the maximizing model predicts that children will choose at chance regardless of the source of the dissenting information, because in either case it is certain that there is one red and one yellow ball. Under the predictions of the probability matching model, children will be at chance when the dissenting information comes from an informant, and will be near chance when provided with their own dissenting information, only slightly preferring their own information as multiple converging sources make it more certain, but not entirely certain, that the majority was provided with a single ball of the opposite colour.

On the other hand, we expected that if children were less sensitive to statistical dependency between social informants than adults, children may view information provided by a majority group that shares a single piece of data to be equivalent to information from a majority group where each member independently collected data. Specifically, we would expect that regardless of the dissenting information, children would endorse the majority in all 4 conditions. In both cases, if children appropriately evaluated their own knowledge as equivalent to that of a single testimony, we would expect that children would show no difference between the dissenting information. If they disproportionately weighted their own evidence, as some prior work has suggested children do, this might result in them being more likely to go against the majority when the child had their own dissenting ball.

### Methods

#### Participants.

A total of 108 preschoolers (female = 54, male = 54; mean age = 58.56 months; range = 48–71 months) were recruited either through local museums or in lab. They were randomly assigned to one of the two dissenting information conditions: the informant (*n* = 48) or the own ball (*n* = 60). Then, within each dissenting information condition, children were then further randomly assigned to one of the two majority ball conditions: independent (informant, *n* = 24; own ball, *n* = 30), or shared (informant, *n* = 24; own ball, *n* = 30).^1^ An additional 10 children were excluded due to experimenter error (8), providing ambiguous answers (1), and inattentiveness (1). Demographic information of participants is provided in the Supplementary Material.

#### Procedure.

In this experiment, children were shown a video on a laptop, where an experimenter introduced two jars comprised of colored balls—one with a 5:1 ratio of yellow to red balls (mostly yellow) and another with a 1:5 ratio (mostly red)^2^—and introduced her three adult friends. Following the procedure used with adults in (Whalen et al., 2018), the experimenter explained that she would pour just one of the two jars into her bag and give each of her friends a cup containing a ball from the bag. To sample a ball, the on-screen experimenter would look up and close their eyes, while appearing to scoop a ball randomly from the bag (the cup in fact already had the desired sample placed in it). They would then hand this cup to the informant. The informants’ balls were never visible to the child. Each informant looked inside their cup and provided testimony as to which jar they thought the bag was filled from, either the jar with mostly red balls in it, or the jar with mostly yellow balls in it. The jar endorsed by the majority and the actors playing the dissenting informant and majority members were counterbalanced in all conditions.

**Figure 1.**
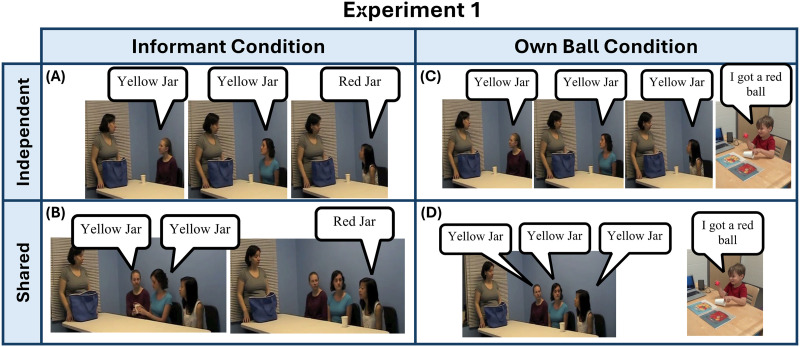
**Task Setup for Experiment 1.** Children viewed 1 of 4 potential videos. In (a), two majority members each received their own independent sample from the bag and each provided their testimony. One informant dissented after receiving their sample. In (b), two majority members were presented with a single sample and each provided their testimony. One informant dissented after receiving their sample. In (c), children viewed three majority members each receiving their own independent sample and each providing their testimony. After viewing the video, the child received a ball of the opposite colour of the testimonies provided by the informants. In (d), three majority members were presented with a single sample and each provided their testimony. After viewing the video, the child received a ball of the opposite colour of the testimonies provided by the informants.

In total, there were 4 conditions given our 2 × 2 design with 2 dissenting information conditions—informant and own ball, and 2 majority ball conditions—independent and shared. In the independent conditions, all informants were in the room one at a time, and were each given their own randomly sampled ball (in a cup) to view (Figure 1a and 1c). After receiving their ball, each informant stated, for instance, “I looked at the ball and I think that the bag has mostly red balls in it.” In the shared data conditions, the members of the majority were all present in the room at the same time and shared a single cup with a single randomly sampled ball (Figure 1b and 1d), which they looked at one at a time. They sat in the same room to emphasize that it was the same piece of data that the majority was sharing. After looking at the ball in the cup and providing their testimony, members of the majority were asked to pass the same cup containing the sampled ball to the next informant, who then first looked in the cup, and then gave their own concurring testimony, until all members of the majority viewed and gave their testimony, e.g., “I looked at the ball and thought about what my friend said. I agree with Jessie. I think that the bag has mostly red balls in it.”

In the dissenting informant conditions, the first two informants always endorsed the same jar and made up the majority group, while the last informant always received their own separately sampled ball and, after looking at it, endorsed the opposite jar, dissenting from the majority (Figure 1a and 1b). For example, if the first two informants said that they thought the balls came from the jar with mostly red balls, the last informant would then receive a different random ball and disagreed, e.g., in the shared data condition, “I looked at the ball and I thought about what my friends said. I disagree with Jessie and Sarah. I think that the bag has mostly yellow balls in it.” On the other hand, in the own dissenting ball conditions, all three informants always endorsed the same jar, while the child would receive a ball opposite of the informants’ testimony (Figure 1c and 1d).^3^ For example, if all informants said that they thought the balls came from the jar with mostly red balls in it, the child would receive a yellow ball. Once the video was completed, the on-site experimenter brought out an identical bag and stated that it was the same bag from the video containing the same balls. She then used an opaque plastic cup to give the child their own ball from the bag. The on-site experimenter pretended to scoop up a ball at random, but in fact the child always received a ball that was a different color from the majority testimony.

Following the informants’ testimony, in all four conditions, the on-site experimenter reminded the child of which jar each informant endorsed, and whether they had seen the same or a different ball as the previous informant, and that all the balls came from just one jar. Finally, children were asked a forced-choice question of which jar the child thought the bag was filled from with the order of the two options randomized.

### Results

**Figure 2.**
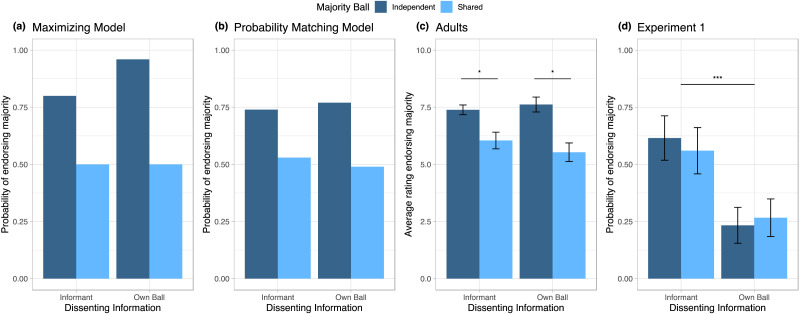
**Endorsement of Majority Testimony by Dissenting Information and Dependency of Testimony.** Maximizing (a) and probability matching (b) Bayesian model predictions for use of majority testimony, and empirical results from adults (c) from Whalen et al. (2018) and from children on Experiment 1 (d). Unlike the predictions of the Bayesian models or previous findings with adults, children were less likely to choose the majority’s testimony when their personal information differed from that of the majority’s testimony, and showed no difference between their choices whether the majority’s testimony was based on independent samples (dark blue) or shared data (light blue).

Each child was given a score of 0 or 1, with 1 representing choosing the jar that aligned with the majority and 0 representing choosing the jar that matched the dissenting information. Results are shown in Figure 2d. To analyze the results, we conducted a logistic regression with the dissenting information condition (informant or own ball) and majority ball condition (independent or shared) as factors.

The analysis revealed that children did not display a sensitivity to statistical dependency; they responded similarly whether the majority’s data was shared or independently sampled, *χ*^2^(1) < 0.001, *p* = .99. However, children’s choices differed depending on the source of the dissenting information; specifically, children were less likely to endorse the majority when they had been given their own dissenting ball compared to when an informant was given a dissenting ball, *χ*^2^(1) = 14.07, *p* < .001. When the dissenting information was provided by an informant, children chose at chance between the two jars, *B* = 0.42, *SE* = 0.30, *OR* = 1.52, 95% *CI* = [0.86, 2.72], *z* = 1.43, *p* = .15. However, when the dissenting information was demonstrated by the child’s own ball, children endorsed the majority significantly below chance, *B* = −1.10, *SE* = 0.30, *OR* = 0.33, 95% *CI* = [0.19, 0.60], *z* = −3.69, *p* < .001.

There was no significant interaction between the dissenting information and majority ball conditions, *χ*^2^(1) = 0.18, *p* = .67. Finally, we found no significant effect of age on children’s choices, and including age as a term in the model did not improve fit, *χ*^2^(1) = 0.0007, *p* = .98.

Notably, these results differ from the choices made by adults in previous work, as well as both the maximization and the probability matching Bayesian models. The probability matching model, which best fit adult performance on the task, predicted a difference of 26% favoring endorsements for the majority over the dissenter across both dissenting information conditions when the informants’ information was from independent sources rather than shared. In contrast, the average difference observed in children was 1.4% in favor of the dissenter when the informants’ information was from independent sources rather than shared.

### Discussion

When comparing children’s choices to those of adults and the predictions of the normative Bayesian models, it appeared that children did not display sensitivity towards statistical dependency, and also differed in how they evaluated the quality of the information provided by different testimony. In both majority ball conditions, children chose similarly, consistent with the hypothesis that children viewed the majority to have information of similar quality, regardless of whether members independently received their own ball or shared the same ball.

However, children did not simply go with the majority in all 4 conditions, as we expected would occur if children were insensitive to statistical dependency. Instead, we found that children’s responses differed based on the kind of dissenting information that was available. When the dissenting information was presented via a dissenting informant, children appeared to choose between the jars at random, although we observed a trend in the direction of choosing the majority group. On the other hand, when the dissenting information was presented as the child’s own ball, children were less likely to endorse the majority’s opinion, suggesting that children may interpret their own ball to be of greater value than that of the majority’s testimony. These results align with previous work in preschoolers’ understanding of causal learning of ambiguous effects in which preschoolers prefer their own interventions other those of others (Kushnir & Gopnik, 2005; Kushnir et al., 2009). Analogously, children in the current study may be biased towards their own collected sample relative to the samples collected by other informants and, consequently, the informants’ testimony.

Additionally, although these two conditions also differed in the number of endorsements provided by the majority—2 in the dissenting informant condition, compared to 3 in the own ball condition—the difference between the model predictions based on the number of majority informants is relatively minor, and it does not account for the difference in children’s choices. Indeed, given that children observed a greater number of endorsements by the majority in the own ball condition, the Bayesian model predicts that children should be slightly more likely to endorse the majority’s opinion in this case, rather than less as we observed.

Alternatively, children choosing at chance in both of the dissenting informant conditions may suggest that they struggled with the complexity of the inferences involved. Although adults succeeded on this task, children may have found it challenging to evaluate the quality of informants’ testimony because they were not sure what the informant saw. For children to follow each piece of evidence, children needed to listen to an informant’s testimony about the contents in the bag, e.g., “I think that the bag has mostly red balls in it,” and infer the ball the informant most likely got from the bag which was filled from one of the two jar options. As a result, given the video’s cognitive demand, children in the own ball conditions may have then decided to ignore the information in the video and rely on their own physical data.

In addition, the child’s own ball was presented live compared to the testimony viewed virtually, creating a difference in salience between the two pieces of evidence. As a result, children may have considered the ball that they received to be a more reliable piece of information. While it has previously been shown that children who encounter evidence that conflicts with an informant will at least sometimes accept the informant’s testimony if the informant is confident and the conflict was probabilistic in nature, they are much less likely to endorse a naive informant when their testimony conflicts with the evidence (Bridgers et al., 2016). Thus, children on our task may have perceived video informants on our task as less reliable in comparison to the concrete evidence that they could see. On top of that, this added further cognitive demand on the children by incorporating not only information from a video but also information presented in-person.

In Experiment 2, we filmed a new set of videos that placed a lower cognitive demand on the children, removing the bag and presenting the child’s own data via video. By simplifying the data presentation and by equating the saliency of all available pieces of evidence by presenting them in the same medium, we aimed to reduce the potential task demands that might have influenced children’s evaluations in Experiment 1.

## EXPERIMENT 2

In Experiment 2, children were shown a similar video to Experiment 1 about two jars with differing proportions. Instead of colored balls, the jars were filled with toys, red fish and green frogs, in the hopes that children found these items more entertaining and were then more attentive. In addition, by having the two items differ in both animal category and color, children might remember and distinguish between the two jar options better. Rather than pouring the contents of the chosen jar into a bag and sampling from it, the on-screen experimenter covered both jars with a black cylinder and sampled directly from the chosen jar. Thus, if children in Experiment 1 struggled to reason about the jars because they could not track how the informants’ samples, which were drawn from the bag, related to the jar that the samples were originally drawn from, a simpler experimental setup where the bag has been removed allows children to directly track the samples being drawn from one of the jars, rather than remembering that a sample from a bag is also the sample from the jar the bag was filled from.

**Figure 3.**
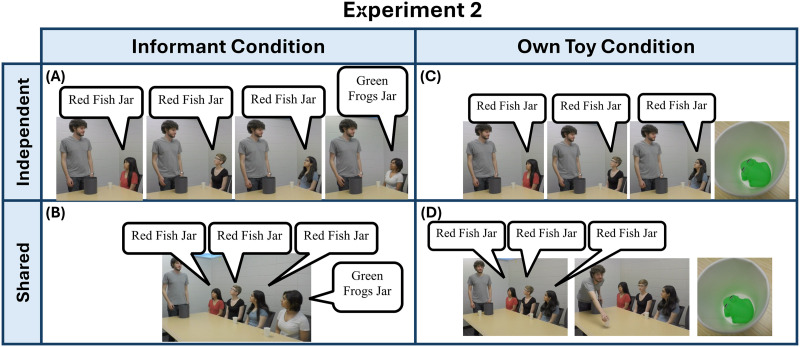
**Task Setup for Experiment 2.** Children viewed 1 of 4 potential videos. In (a), three majority members each received their own independent sample from the bag and each provided their testimony. One informant dissented after receiving their sample. In (b), three majority members were presented with a single sample and each provided their testimony. One informant dissented after receiving their sample. In (c), children viewed three majority members each receiving their own independent sample and each providing their testimony. After viewing the video, the child received a toy of the opposite colour of the testimonies provided by the informants. In (d) , three majority members were presented with a single sample and each provided their testimony. After viewing the video, the child received a toy of the opposite colour of the testimonies provided by the informants.

In Experiment 1, the total number of informants was equated between groups—there were always three informants. However, as a result, the majority size differed between conditions, with a three informant majority in the own ball conditions, and a two person majority in the dissenter conditions. In Experiment 2, the majority group always consisted of three members. In this way, we could then better directly compare between the dissenting informant and own toy conditions and how children evaluated a majority with the same number of informants and testimony; additionally, this change increased the difference between independent and shared data in the dissenting informant condition predicted by the Bayesian model, making it easier to determine whether children in this condition were sensitive to statistical dependency (Figure 3).

Lastly, in the own ball conditions, once the three informants provided their testimony, the on-screen experimenter would sample a toy from the chosen jar in the same manner as they did for the informants’ samples, then show the child their sample via video. In this way, we reduced the potential for confusion resulting from the differing salience of the child’s own real world 3D sample relative to the video testimony, that may have been present in Experiment 1. If children in Experiment 1 put additional weight on their own ball because of its salience as a real-world object, then this might reduce the potential strength of a “self-agency bias” that might have driven children’s strong reliance on their own ball when it was available, and thus result in a reduced tendency to endorse the jar consistent with their own data, rather than the jar endorsed by the majority.

**Figure 4.**
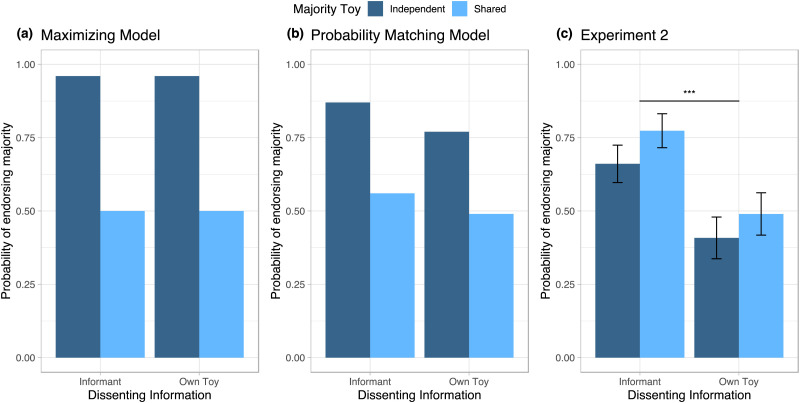
**Endorsement of Majority Testimony by Dissenting Information and Dependency of Testimony.** Maximizing (a) and probability matching (b) Bayesian model predictions for use of majority testimony, and empirical results from children on Experiment 2 (c). Unlike the predictions of the Bayesian models, children were less likely to choose the majority’s testimony when their personal information differed from that of the majority’s testimony, and showed no difference between their choices whether the majority’s testimony was based on independent samples (dark blue) or shared data (light blue).

In sum, we minimized the task demands related to tracking the evidence that informants have observed, increased the size of the majority group in the dissenting informant condition so that there were always three informants in the majority group, and reduced the salience of the child’s own data. In doing so, we anticipated that if children were reasoning about statistical dependency in the same way as adults, that they would choose jars at chance when the majority shared a single data point to base their testimony off of, and choose the jar endorsed by the majority when the majority’s testimony was supported by independent data points, similarly to the predictions made by the idealized model (Figure 4).

### Methods

#### Participants.

Given our surprising results in Experiment 1, we also wanted to investigate a possible age effect between the 4- and 5-year-old children. As such, we doubled our sample size and collected an equal number of 4- and 5-year-old children per condition. A total of 192 preschoolers (female = 93, male = 97, genderless = 2; mean age = 59.70 months; range = 48–71 months) were recruited through local museums, public parks, and a lab database. They were randomly assigned to one of the two dissenting information conditions: the informant (*n* = 96) or the own toy (*n* = 96). Then, within each dissenting information condition, children were then further randomly assigned to one of the two majority toy conditions: independent (informant, *n* = 48; own toy, *n* = 48) or shared (informant, *n* = 48; own toy, *n* = 48). An additional 36 children were excluded due to inattentiveness towards the video (19) such as removing their headphones, talking over the video, or outside distractions, experimenter error (11) such as mislabeling the informants and/or their testimony, and providing ambiguous answers (6) such as switching their response with or without prompts or picking both jars. Demographic information of participants is provided in the Supplementary Material.

#### Procedure.

The procedure was largely similar to Experiment 1, with the following changes:

- To increase both the interest of the stimuli and the difference between the two options, instead of colored balls, the jars were comprised of red fish toys and green frog toys—one with mostly red fish toys and a few green frog toys, and one with mostly green frog toys and few red fish toys.
- Instead of a bag, the experimenter explained that he would cover both jars and just pick only one of his two jars to scoop toys from and give to his friends.
- To make the statistical reasoning in both conditions more similar, the size of the majority in the dissenting informant condition was made equal to that of the own toy condition (3).
- The child’s toy was sampled and shown to the child via video in the same way as the informants’ samples to minimize potential saliency effects.
- In all four conditions, once the video was completed, the on-site experimenter reminded the child which informant endorsed which jar, if they looked at the same or different toy as the previous informants, the child’s own toy if applicable, and that all the toys came from just one jar.
- Children were asked an open-choice question of which jar the child thought was chosen. If children were hesitant in answering, children were given a follow-up forced-choice question asking which jar, either the jar with mostly red fish or green frogs in it.

### Results

As in Experiment 1, each child was given a score of 0 or 1, with 1 representing choosing the jar that aligned with the majority and 0 representing choosing the jar that matched the dissenting information. Results are shown in Figure 4c. We again conducted a logistic regression with the dissenting information condition (informant or own toy) and majority toy condition (independent or shared) as factors.

Similarly to Experiment 1, and unlike the predictions of the idealized model, we found that children were not sensitive to statistical dependency in the majority’s testimony, as they did not respond differently when the majority’s data was shared rather than independently sampled, *χ*^2^(1) = 1.49, *p* = .22. On the other hand, they were less likely to endorse the majority when they had been given their own dissenting ball, compared to when an informant was given a dissenting ball, *χ*^2^(1) = 14.81, *p* < .001. Once again, no interaction was found between the dissenting information condition and the majority toy condition, *χ*^2^(1) = 0.19, *p* = .66. Unlike Experiment 1, children endorsed the majority significantly above chance levels when the dissenting information was provided by a dissenting informant (*B* = 0.95, *SE* = 0.23, *OR* = 2.58, 95% *CI* = [1.65, 4.03], *z* = 4.15, *p* < .001); when the dissenting information was the child’s own ball, they were at chance between jars (*B* = −0.21, *SE* = 0.21, *OR* = 0.81, 95% *CI* = [0.54, 1.21], *z* = −1.02, *p* = .31). A model including age as a term found a non-significant, but marginal effect of age, with older children choosing the majority marginally more often, *χ*^2^(1) = 3.38, *p* = .07.

In both Experiments 1 and 2, we observed a similar pattern: children were more likely to endorse the majority in the dissenting informant condition compared to the own data condition, and were equally likely to endorse the majority whether the majority’s information was independent or shared. However, as these conditions resulted in differing significance relative to chance, we conducted an exploratory analysis combining data from Experiments 1 and 2.

Consistent with a reduced bias towards their own data, children were more likely to endorse the majority in Experiment 2 than Experiment 1, *χ*^2^(1) = 6.96, *p* = .008. As with both prior analyses, we found that children did not respond differently whether the majority’s data was shared or independently sampled, *χ*^2^(1) = 1.02, *p* = .31, but that they were less likely to endorse the majority when they had been given their own dissenting toy compared to when an informant was given a dissenting toy, *χ*^2^(1) = 28.67, *p* < .001. There was also a significant main effect of age, with older children slightly more likely to endorse the majority than younger children, *χ*^2^(1) = 4.33, *p* = .038.The analysis revealed no significant interactions (all *χ*^2^(1) < 0.52, all *p* > .47), suggesting that while children may have found their own data more salient in Experiment 1 than Experiment 2, children evaluated the quality of informants’ information and integrated their own data similarly across both experiments. Supporting this interpretation, when the experiments were analyzed together, children endorsed the majority significantly above chance when the dissenting information was provided by a dissenting informant (*B* = 0.76, *SE* = 0.18, *z* = 4.24, *p* < .001), but were significantly below chance when the dissenting information was the child’s own ball (*B* = −0.53, *SE* = 0.17, *z* = −3.17, *p* = .002).

Lastly, we collected open-ended explanations that children provided for their choices on the task, and two experimenters (one blinded to the hypotheses of the experiment) coded them according to whether they referred to the majority’s testimony, the minority’s testimony (or the child’s own data, when the dissenting information was the child’s own ball), the experimenter in the video, the distribution of the toys in the jar, their own preferences, or irrelevant considerations. Interrater agreement was high (Cohen’s *κ* = 0.82); disagreements were resolved through discussion.

We found that across the dissenting information conditions, children’s justifications differed substantially (*χ*^2^(6) = 58.03, *p* < .001); when the child received personal conflicting information, they referred to the minority information (e.g., “my cup had a frog”) more often, *e*_*ij*_ = 6.73, *p* < .001, and referred to the majority information (e.g., “more people said it was in the frogs jar”) less often, *e*_*ij*_ = −3.10, *p* < .001. Conversely, we did not find a significant difference in children’s explanations across the majority conditions (Independent vs. Shared), *χ*^2^(6) = 6.52, *p* = .367. These explanations matched the similar pattern of results observed for children’s endorsement choices.

### Discussion

In comparison to the *a priori* predictions of the idealized Bayesian models, we again found that children were no more likely to endorse the majority group when the members independently collected data compared to when they shared a single piece of data, suggesting that children evaluate information quality differently to that of adults, and may be predisposed to follow the group with the greatest number of endorsements when given testimony alone. However, they were not simply showing a conformity bias; across both Experiments 1 and 2, children were significantly less likely to endorse the majority when their own data differed from the informants’, relative to when the dissenting information was provided by another informant. This may indicate that children consider their own data a more reliable or important source of information.

Additionally, when combining data from Experiments 1 and 2, we found that older children were slightly more likely across conditions to endorse the majority than younger children. This mirrors a similar developmental trajectory observed in Morgan et al. (2015), in which older children and adults exhibited stronger conformity to non-total majorities than younger children.

One possibility for the difference between our 2 dissenting information conditions, and between children’s performance in Experiments 1 and 2 and adults’ previous performance on a nearly identical task (Whalen et al., 2018), may be that at this age, children have difficulty inferring the likely identity of the hidden data the informants’ received based on their testimony alone, but are likely confident in what they themselves see—in this case, their own data. As a result, in the dissenting informant conditions, where children had no direct access to data, children may have found it difficult to infer what was most likely in the informants’ cups, but nonetheless reasoned that three endorsements were better than one (regardless of the data they were based on). In contrast, in the own dissenting toy conditions, children may have preferred their own visible data to the unknown contents of the informants’ cups, being unable to infer what was likely inside them.

While 4- and 5-year-olds understand that others can have different mental states and perceptual access from their own, an understanding of the information quality in our experiment may require more complex epistemic reasoning that continues to develop between ages 4 and 6 (Aboody et al., 2022). Here, children may be struggling to infer the sample that an informant encountered given that informant’s subsequent testimony. Understanding statistical dependency in our experiment requires children to not only track the number of testimonies, but also infer the data that the informants likely received to produce this testimony. For example, if an informant said, “I looked at my toy and I think the jar has mostly green frogs in it,” the child must be able to reason that the informant most likely saw a green frog. If this inference was difficult for children, they may have relied on the number of informants instead when no certain data was available. This may explain why children responded similarly regardless of the statistical dependency of the majority’s information in Experiments 1 and 2, as well as why children were more likely to choose the jar consistent with the majority’s choice in Experiment 2, since the child could not rely on their own visible evidence.

Another possibility is that children were able to infer what samples the informants likely received, but were not able to reason statistically about the informants’ samples, in order to make an inference back to the population the samples were likely drawn from. In other words, even if children were able to correctly infer which toy each informant likely saw, they might still have failed to understand how to integrate this evidence to draw a probabilistic conclusion about the jar the toys were likely taken from.

Although children at this age can reason about the likely composition of populations given a set of samples (e.g., Denison et al., 2006), it is possible that our task or sampling method made this difficult. To ensure that children’s response in Experiments 1 and 2 reflected differing evaluations of the value of their own and the majority’s information, rather than difficulties with reasoning about the probability of the samples, we conducted a third experiment with a video that removed the informants and only presented the samples, which were all fully observable to the child. If children’s performance in Experiments 1 and 2 reflects difficulty in inferring samples from testimony, we would expect children’s choice of jar in Experiment 3 to change depending on whether the samples are independent or shared, more closely matching the predictions of the Bayesian rational model. Additionally, if children’s relative preference for their own toy in Experiments 1 and 2 reflects a naïve bias towards their own information, above and beyond difficulties with reasoning about what other informants had likely seen, we would expect children in this experiment to show a stronger preference towards choosing the jar consistent with their own toy in the own toy condition.

## EXPERIMENT 3

**Figure 5.**
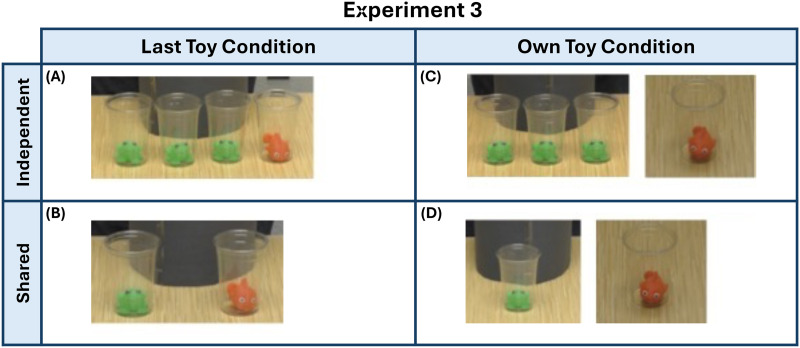
**Task Setup for Experiment 3.** Children in Experiment 3 viewed 1 of 4 potential videos. In (a), the “majority samples” were represented by the first three toys while the final toy differed; all samples were presented together. In (b), the “majority sample” was represented by the first toy; the second toy differed, and was presented with the first toy. In (c), the “majority samples” were represented by the first three toys; the last sample differed and was presented far away from the other toys. In (d), the “majority sample” was represented by the first toy; the second toy differed and was presented far away from the other toy.

In this experiment, children were shown a video introducing the same two jars of toys as seen in Experiment 2. Unlike Experiment 2, the on-screen experimenter sampled the chosen jar using clear cups, instead of the opaque ones, making the toy samples visible for the child to see. Informants were no longer present. Instead, the clear cups contained a toy that corresponded to the most likely toy that the informants would have seen. For example, in the independent dissenting information condition, the on-screen experimenter would now sample, three clear cups containing green frogs, and one clear cup containing a red fish (see Figure 5).

### Methods

#### Participants.

A total of 192 preschoolers (female = 99, male = 93; mean age = 59.41 months; range = 45–71 months) were recruited through local museums. They were randomly assigned to one of two dissenting information conditions: the last toy (*n* = 96) or the own toy (*n* = 96). Then, within each dissenting information condition, children were then further randomly assigned to one of the two majority toy conditions: independent (last toy, *n* = 48; own toy, *n* = 48) or shared (last toy, *n* = 48; own toy, *n* = 48). An additional 13 children were excluded due to providing no answers or ambiguous answers (5), inattentiveness (4), experimenter error (3), or did not complete the experiment (1). Demographic information of participants is provided in the Supplementary Material.

#### Procedure.

The stimuli and procedures were similar to that of Experiment 2, however, no informants were present within the video. The video began with the on-screen experimenter introducing the same two jars used in Experiment 2, explaining that he would cover both jars and pick just one jar to sample from. Unlike Experiment 2, the on-screen experimenter sampled from the chosen jar using clear cups so that the samples from the chosen jar were visible to the child. Within each clear cup, a single toy was inside, either a red fish or green frog toy. The number of clear cups used to sample was equivalent to the number of opaque cups in Experiment 2, to represent the same amount of data. The toy inside the cup would also reflect the likely toy the informants saw in Experiment 2. The order in which the samples were taken remained the same, mimicking how the majority were always taken out first and the dissenting sample was taken out last.

As in the previous experiments, children were randomly assigned to one of four possible conditions within our 2 × 2 design with 2 dissenting information conditions—last toy and own toy, and 2 majority toy conditions—independent and shared. In the last toy conditions, all sampled cups were placed in the front of the jar together and the last toy sampled was always different to that of the ‘majority’ sample (Figure 5a and 5b). On the other hand, in the own toy conditions, as in Experiment 2, once the ‘majority’ toys were sampled and placed in front of the jar together, the experimenter would state that the last cup would be the child’s toy and placed it away from the rest (Figure 5c and 5d). The ‘majority’ conditions represent the number of initial samples: three (independent) or one (shared). For clarity, we refer to these as the ‘majority’ toy conditions, with the same names as those used in Experiments 1 and 2; however, as there are no longer any informants, there is no numerical majority in the shared condition, as each toy is only sampled once.

### Results

**Figure 6.**
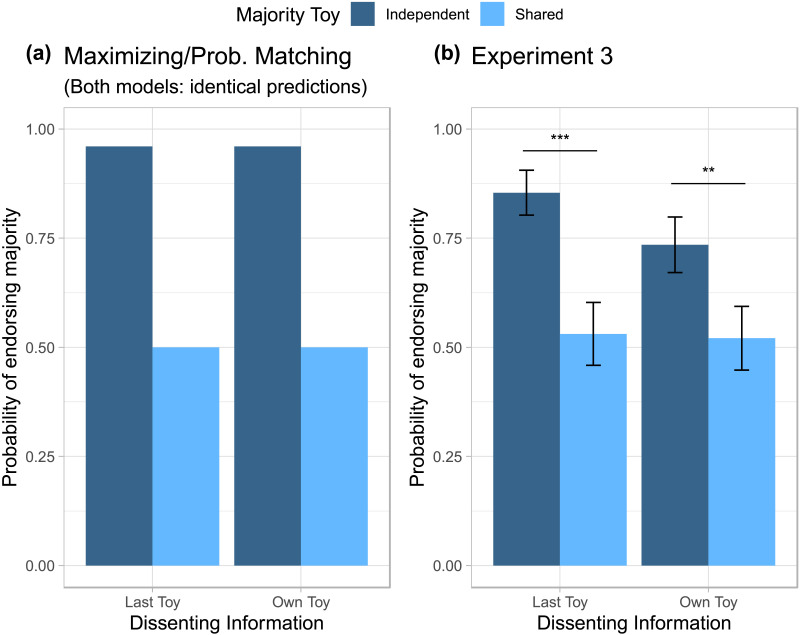
**Endorsement of Majority Jar by Dissenting Information and Dependency of Samples.** Maximizing Bayesian model predictions (a) and empirical results from children on Experiment 3 (b). As no testimony was involved in Experiment 3, the predictions for the probability matching model are equivalent to the maximizing model. Unlike Experiments 1 and 2, children’s choices were qualitatively similar to the Bayesian model predictions, choosing the majority toy more often when the majority sample was independently drawn three times, and choosing at chance when the majority sample was shared. Children also did not show a significant preference for their own toy.

Each child was given a score of 0 or 1, with 1 representing choosing the jar that aligned with the first toy or the ‘majority’ and 0 representing choosing the jar that matched the final toy or the dissenting information. We again conducted a logistic regression with the dissenting information condition (last toy or own toy) and majority ball condition (independent or shared) as factors. Results are shown in Figure 6b. In line with the predictions of the Bayesian model, children in Experiment 3 were more likely to endorse the majority when the majority information was independent than when it was shared, *χ*^2^(1) = 17.42, *p* < .001. However, they were no more likely to endorse either jar when the toy was the last toy or the child’s own toy, *χ*^2^(1) = 1.21, *p* = .27, and there was no interaction, *χ*^2^(1) = 1.19, *p* = .28. Regardless of the nature of the dissenting information, children chose the majority significantly above chance when the majority information was independent, *B* = 1.47, *SE* = 0.26, *OR* = 4.31, 95% *CI* = [2.58, 7.25], *z* = 5.57, *p* < .001, and were at chance between jars when the majority was shared, *B* = 0.13, *SE* = 0.21, *OR* = 1.13, 95% *CI* = [0.76, 1.70], *z* = 0.61, *p* = .54. In other words, children favored choosing the green frog jar or red fish jar when there were more green frog toys or red fish toys, respectively, and were at chance between the jars when there was one of each toy. As in Experiment 2, we found a non-significant, albeit marginal effect of age, with older children choosing the majority marginally more often, *χ*^2^(1) = 3.26, *p* = .071.

### Discussion

Children correctly inferred the probability that the samples were drawn from a particular population regardless of the nature of the dissenting information, and their responses were qualitatively similar to the predictions of the *a priori* optimal Bayesian model. Even though the Bayesian model makes identical predictions to the maximizing model in Experiment 2, children in Experiment 3 did not exhibit the same insensitivity to the majority toy conditions that they did in Experiment 2. Given that children showed no significant difference between the dissenting information conditions, we can also conclude that children do not simply demonstrate a nave bias towards their own toy as, when the samples were visible rather than inferred from testimony, they treated their own sample equally to that of any other sample drawn from the jar.

Taking these results together with the findings of Experiments 1 and 2, we suggest that children may have exhibited a bias towards their own data, as well as an insensitivity to statistical dependency, due to their difficulty with inferring the samples others likely received from their testimony. In the absence of informants, and consequently testimony, children were able to track the number of samples and rationally infer the identity of the chosen jar based on the distribution of toys removed from the jar. Yet, once the samples were not visible to them, children may have struggled to infer informants’ likely samples, preferring to make decisions based on facts that they were certain of, such as the number of endorsements or the nature of their own toy.

## GENERAL DISCUSSION

In this study, we investigated 4- and 5-year-old children’s sensitivity to statistical dependency in testimony. We found that, although children were able to reason about the statistics of the task when no testimony was present, children systematically preferred testimony from a larger group to testimony from a dissenting informant, and preferred their personal data to evidence that had to be inferred from testimony. These results differed from the predictions of the Bayesian rational models as well as from adults’ performance in prior research, suggesting that reasoning about the role of independent versus shared evidence in social testimony is still developing at 4 and 5 years of age.

In Experiments 1 and 2, we found that children responded similarly regardless of whether the majority’s data was independently collected or shared. Surprisingly, we also observed that children’s inferences greatly differed based on how the dissenting information was presented, in that children were less likely to endorse the majority when they received their own data compared to when there was a single dissenter, and in fact tended to favored their own data over that provided by even an independent majority. We hypothesized that these responses may have been the result of children struggling to infer the identity of the hidden samples most likely received by each informant based on their testimony alone and, as a result, relying on information they were certain of, such as the number of endorsements for each option, and the identity of their own toy.

In Experiment 3, we removed informants from the video and explicitly displayed the evidence that would have been inferred in the conditions of the previous experiments. Similarly to the predictions of the Bayesian model, children were able to predict the probability that the “majority” jar was chosen given all the samples observed, regardless of whether the toys belonged to the child or not. From this we concluded that children are likely not simply naively preferring their own data, but instead were likely relying more heavily on the information that they were certain of, and underweighting or ignoring the probability of uncertain or unknown data, such as the hidden data that informed the testimony by informants in Experiments 1 and 2.

Although even infants are capable of probabilistic reasoning (Denison, Reed, & Xu, 2013; Denison & Xu, 2010), children’s representation of possibility and uncertainty develops substantially over the first few years of life (Leahy & Carey, 2020). For example, when children are shown two candidate toys that might be inside a box, but not which one was put inside, children under age 6 mistakenly consider their guess to be equivalent to knowledge (Rohwer et al., 2012). Thus, children on our task may have found it difficult to construct multiple different counterfactual possibilities for what the informants had likely observed, and as a result, considered the contents of the informants’ cups generally unknown, rather than inferring the (still uncertain, but more precise) estimated probabilities that each toy would be in an informant’s cup). Thus, in both dissenting information conditions, children may have relied on information that they were certain about: when children did not directly observe any data, they could be certain about the number of informants endorsing each jar; conversely, when children directly observed a piece of evidence, they could be certain about the evidence that they themselves received. Alternatively, studies within causal domains have observed that children rely on their own actions over the actions of others for the cause of an ambiguous or probabilistic effect (Kushnir & Gopnik, 2005; Kushnir et al., 2009). Children in these situations may view their own actions to be more controlled and reliable and less likely to be confounded than those of other individuals. Children may find it challenging to understand the reasoning behind the actions of others in a probabilistic setting and as a result, reasonably trust their own actions. This preference for one’s own data has not previously been observed in a non-causal domain, such as that of the current study. Future work should consider which situations may prompt children to prefer their own data over others within a non-causal domain.

The difficulty of inferring the data others may have observed, leading to their testimony or actions, may reflect ongoing development in theory of mind (e.g., Wellman et al., 2001) Although 4- and 5-year-old children know that people can have beliefs that differ from their own, and that perceptual access provides them with knowledge (e.g., Butler et al., 2018; Pillow, 1989), the task of inferring what someone else was likely to know or believe based on their subsequent behavior may be more challenging for children at this age (Aboody et al., 2019; Wu & Schulz, 2018, but see Jara-Ettinger et al., 2017). In combination with findings from previous work (e.g., Aboody et al., 2022; Einav, 2018), our work suggests that a clear understanding that independent, converging testimony is more informative than a single piece of testimony emerges around age 6, and more complex theory of mind abilities continue to develop throughout middle childhood alongside greater vigilance towards more nuanced forms of informativeness such as deception and distortion (Mills & Elashi, 2014; Tay et al., 2024). Thus, although children in our task may have struggled to reason about what informants had likely observed, they were likely confident in the number of endorsements made by informants as well as in what they themselves saw. Thus, another avenue for future research is to consider whether a developed theory of mind facilitates the developmental trajectory in evaluating statistical dependencies in social learning.

When combining data from Experiments 1 and 2, we also observed a small age effect, in which older children were more likely to endorse the majority across all conditions. One reason for this may be that in the dissenting informant condition, the majorities observed in our task were non-total majorities. Younger children have been shown to be less sensitive to the endorsements of non-total majorities endorsing something than older children (Morgan et al., 2015).

It is also possible that, at least in the absence of their own conflicting information, children may show the same susceptibility to the “illusion of consensus” observed in adults (Desai et al., 2022; Yousif et al., 2019). Although adults’ performance on a similar task to the one we conducted with children closely resembled the predictions of a normative Bayesian model (Whalen et al., 2018), the circumstances under which testimony is statistically dependent may be less obvious to children, and children may need to be older to understand the flow of information on a task where they must track multiple informants with multiple different potential knowledge sources (e.g., Aboody et al., 2022; Einav, 2018).

Alternatively, children may also make different pragmatic assumptions about the significance of statistical dependence when they encounter dependent testimony. For example, young children may see dependent testimony as more reliable than independent testimony because they observe dependent informants explicitly agreeing with one another (Einav, 2018); they may also see dependent informants attending to another informant as a cue to the prestige and thus potential reliability of the informant (Chudek et al., 2012). Although not significant, we did observe that children on our task exhibited a weak trend consistent with this, conforming to the dependent majority more than the independent majority in Experiments 1 and 2. Increasing the salience of the statistical dependency exhibited by the informants on our task, e.g., by highlighting to children the number of sources each group’s testimony is based on, may make it easier for younger children to reason about, or provide clarity as to whether children draw different conclusions about statistical dependency from adults.

Lastly, we want to note limitations to generalization from this sample. Participants in this set of studies were recruited from a large, multicultural Canadian city, with a comparable ethnic diversity to the city as a whole (see Supplementary Material for a full breakdown). Nevertheless, a full understanding of children’s evaluation of the statistical dependency in social learning requires consideration of the differing ways that testimony is understood across cultures. For example, lower levels of dissension and greater deference to the testimony of majorities have been found among adults from collectivistic countries than individualistic ones (e.g., Bond & Smith, 1996); similarly, children of East Asian descent were more receptive to consensus information than European American children (Corriveau & Harris, 2010; Corriveau et al., 2013; DiYanni et al., 2015). Notably, cultural differences may also reflect differing attitudes towards informants based on their status or characteristics. For example, Chinese children were more willing to endorse a consensus of adults than Spanish children (Enesco et al., 2016), but Spanish children were more willing to endorse a consensus of peers than Chinese children (Sebastián-Enesco et al., 2020). Thus, whether an informant is perceived as a learning authority may substantially change how children from different cultural backgrounds approach their testimony.

### Conclusion

Taken together, our findings show that 4- to 5-year-old children do not evaluate statistical dependency in social testimony in the same way as adults, appearing to mistakenly place greater weight on statistically dependent testimony from a majority and their own sampled data than the predictions of an idealized rational model. Rather than reflecting a nave bias to conform to a majority, or to prefer their own data, we suggest that children’s performance on this task reflects their difficulty with inferring the data available to an informant based on testimony alone. Faced with such uncertainty, children may rely upon the information they feel more confident about, such as their own personal data or the number of informants available to learn from, when making decisions about whose version of a story to trust.

These findings provide important insight on how children integrate social testimony with their own knowledge in a world filled with uncertainty. Attending disproportionately to the most frequently encountered information or behavior has been suggested as a common social learning strategy for humans and a number of other species (Kendal et al., 2018). Further, while witnessing an informant endorsing the same hypothesis as another without obtaining additional data may not provide more information from an epistemic standpoint, this may serve as a signal to children of the initial informant’s prestige and thus potential credibility (e.g., Chudek et al., 2012). These may be particularly useful strategies for young children, who are growing up in communities with many normative as well as epistemic considerations (Gelpí & Buchsbaum, 2024). By treating information from majorities as inherently informative, but also placing additional value on one’s own information, children may be able to effectively balance learning from others and from their own exploration as they develop.

## FUNDING INFORMATION

This research was supported by a SSHRC Insight Grant to DB.

## AUTHOR CONTRIBUTIONS

K.O., A.W., and D.B. conceived and designed the experiments. K.O. conducted the experiments, R.A.G., K.O., A.W., and D.B. conducted formal analysis of the experiments, R.A.G. validated the analysis and created the visualizations, and D.B. supervised the research. R.A.G. and K.O. wrote the original draft, and D.B. reviewed and edited the draft.

## DATA AVAILABILITY STATEMENT

Data and analysis code are available at https://osf.io/z3tj8/.

## Notes

^1^ As part of our agreement with the local museums where the experiment was conducted, all children were allowed to participate in the studies offered on a given day. As a result, an additional 12 children were run who were included within the analysis. Within the Supplementary Material, an alternative analysis excluding these participants demonstrated no difference to our findings.^2^ Each jar also included one distractor ball of a different colour (one green ball in the mostly yellow jar, one white ball in the mostly red jar). The distractor balls were visible to the children, and were factored into the predictions derived from our models, but were never sampled during the experiment.^3^ The number of informants differed between the two dissenting information conditions (3 in the own ball conditions and 2 in the dissenting informant conditions) as we wanted to keep the number of total informants consistent across conditions, as well as to mirror the experimental conditions completed by adults in Whalen et al. (2018), which used this same design. In Experiment 2, all experimental conditions included a majority group of 3 people.
